# A 3-Year Prospective Study on a Porcine-Derived Acellular Collagen Matrix to Re-Establish Convexity at the Buccal Aspect of Single Implants in the Molar Area: A Volumetric Analysis

**DOI:** 10.3390/jcm9051568

**Published:** 2020-05-22

**Authors:** Célien Eeckhout, Eline Bouckaert, Dagmar Verleyen, Thomas De Bruyckere, Jan Cosyn

**Affiliations:** 1 Faculty of Medicine and Health Sciences, Oral Health Sciences, Department of Periodontology and Oral Implantology, Ghent University, Corneel Heymanslaan 10, B-9000 Ghent, Belgium; Eline.Bouckaert@Ugent.be (E.B.); Dagmar.Verleyen@Ugent.be (D.V.); Thomas.DeBruyckere@Ugent.be (T.D.B.); Jan.Cosyn@Ugent.be (J.C.); 2 Faculty of Medicine and Pharmacy, Oral Health Research Group (ORHE), Vrije Universiteit Brussel (VUB), Laarbeeklaan 103, B-1090 Brussels, Belgium

**Keywords:** collagen matrix, soft tissue substitute, soft tissue augmentation, dental implant, single tooth

## Abstract

Background: Xenogeneic soft tissue substitutes are currently being investigated as an alternative to subepithelial connective tissue grafts (CTG) with the intention to avoid postoperative morbidity associated with autologous grafting. The aim of the present study was to volumetrically evaluate the effectiveness and mid-long-term stability of a porcine-derived collagen matrix (PDCM) (Mucoderm^®^, Botiss gmbh, Berlin, Germany) in increasing soft tissue volume at the buccal aspect of molar implant sites. Methods: Periodontally healthy non-smoking patients with a single tooth gap in the molar area were selected for a prospective case series. All sites had a bucco-oral bone dimension of at least 8 mm and demonstrated a horizontal alveolar defect. A wide diameter implant was placed under the elevated buccal flap and a PDCM was applied. The primary outcome was the linear increase in buccal soft tissue profile (BSP) within a well-defined area of interest. This was performed with designated software (SMOP; Swissmeda AG, Zurich, Switzerland) on the basis of superimposed digitalized study casts taken before surgery (T0), immediately after surgery (T1), at three months (T2), one year (T3) and three years (T4). Secondary outcomes were alveolar process deficiency and clinical parameters. Results: Fourteen out of 15 treated patients attended the three-year re-assessment (four females; mean age 51.4 years). Mean linear increase in BSP at T1 was 1.53 mm (*p* = 0.001). The PDCM showed substantial resorption at T2 (1.02 mm or 66.7%) (*p* = 0.001). Thereafter, a 0.66 mm volume gain was observed (*p* = 0.030), possibly due to the installation of a permanent crown displacing the soft tissues to the buccal aspect. This resulted in a linear increase in BSP of 1.17 mm (76.5%) at T4. Alveolar process deficiency significantly reduced over time (*p* = 0.004). However, 50% of patients still demonstrated a slight (6/14) or obvious (1/14) alveolar process deficiency at study termination. Implants demonstrated healthy clinical conditions. Conclusions: The PDCM demonstrated marked resorption during the early stages of healing. Due to the matrix thickening the tissues, and the permanent crown displacing the tissues, 76.5% of the initial increase in BSP could be maintained over a three-year period. Half of the patients failed to show perfect soft tissue convexity at the buccal aspect.

## 1. Introduction

The success of dental implant treatment is no longer solely defined by successful osseointegration and implant function over time. Patients have become more demanding with respect to aesthetic aspects. As a result, soft tissue augmentation procedures are often performed at implant sites to improve the aesthetic outcome [1]. This seems also to be justified from a biological point of view, since significant buccal bone remodeling and thus loss of buccal convexity are inevitable following tooth extraction [2]. The latter may hamper a favorable aesthetic result as a horizontal volume loss at the buccal aspect causes a shadow in the corresponding region [3]. Therefore, soft tissue augmentation can be performed to partially compensate for missing volume at the buccal aspect of implants. This can only be considered if the remaining bone volume allows complete embedding of the implant shoulder [4,5,6,7]. The critical threshold value of mucosal thickness from an aesthetic point of view appears to be 2 mm. In vitro and in vivo data suggest that when the mucosal thickness exceeds 2 mm, the human eye is incapable of detecting any discoloration of the mucosa due to underlying restorative materials [8,9]. From a biological point of view, however, there is still a lack of scientific evidence as to whether thicker peri-implant soft tissues limit the incidence of peri-implant disease [10]. According to a recent systematic review, the use of a subepithelial connective tissue graft (CTG) for gain of mucosal thickness resulted in significantly less marginal bone loss over time but no significant improvement in bleeding on probing, pocket depth and plaque index [11].

The use of CTG harvested from the palate is considered the gold standard for soft tissue augmentation around dental implants [6,7]. Long-term data are available showing 85% relative horizontal stability at the buccal aspect after five years [12]. However, its greatest demerit is the harvesting procedure at the donor site, which may lead to complications such as palatal bleeding, swelling, infection or necrosis [6,13,14]. In addition, anatomical and individual limitations exist, leading to a variable quantity and quality of tissue that can be harvested [15]. Therefore, current research focuses on alternative devices to replace autogenous tissue.

A newly developed porcine-derived acellular collagen matrix (PDCM) (Mucoderm^®^, Botiss gmbh, Berlin, Germany) composed of non-crosslinked natural type I and type III collagen was introduced and found to be successful for gingival augmentation and root coverage [16,17,18,19,20,21,22,23]. More recently, the matrix was proposed as an autogenous graft substitute for increasing soft tissue thickness under closed healing conditions. The ability to increase soft tissue thickness with good histological integration was demonstrated in preclinical studies [24,25,26,27,28,29]. To the best of our knowledge, two clinical studies have been performed with this matrix, yet both demonstrate important limitations [23,30]. First, there was no proper baseline registration in these studies. Second, measurements were performed using transmucosal probing by an endodontic instrument or anesthesia needle with a silicone stop. Both may be considered quite inaccurate when small tissue changes are to be identified. Finally, the follow-up of these studies ranged between 6 and 12 months.

The primary objective of this three-year prospective study was to volumetrically evaluate the effectiveness of this PDCM in increasing buccal soft tissue volumes at single implants in the molar area.

## 2. Material and Methods

### 2.1. Patient Selection

Patients in need of a single implant restoration in the molar area were enrolled in a private practice and at the University Dental Clinic of Brussels University (VUB) between 2015 and 2017 to be involved in a prospective case series. Patients were recruited according to inclusion and exclusion criteria.

Inclusion criteria were as follows:

- Minimum 18 years old
- Good oral hygiene (Full-mouth plaque score ≤ 25%) [31]
- A single tooth gap in the molar area
- Adjacent tooth present at both sides of the defect
- Failing tooth at least 3 months prior to enrolment removed
- Seibert Class I defect at the single tooth gap as clinically assessed (buccal concavity with a normal apicocoronal ridge height) [32]
- Sufficient amount of keratinized mucosa available at the single tooth gap
- Bucco-oral bone dimension of at least 8 mm as assessed on cone beam computed tomography (CBCT) at the central and crestal aspect of the single tooth gap to ensure complete embedding of a wide diameter implant by bone
- Signed informed consent

Exclusion criteria were as follows:

- Systemic diseases
- Smokers
- Untreated caries lesions
- (History of) periodontal disease
- Horizontal bone augmentation required at implant placement

The PROCESS statement for preferred reporting of case series in surgery was adopted [33,34]. The study was approved by the Ethical Committee of the Brussels University Hospital (UZ Brussel; B.U.N. 143201524133) and performed in accordance with the ethical standards of the Declaration of Helsinki in 1975, as revised in 2013.

### 2.2. Surgical and Prosthetic Procedures

Patients were instructed to take systemic antibiotics (Amoxicillin 2 g) and anti-inflammatory medication (Ibuprofen 600 mg) 1 h pre-operatively. Just prior to the treatment, patients rinsed with a 0.12% chlorhexidine solution (Perio-aid^®^ Intensive Care, Dent-Aid Benelux, Houten, The Netherlands).

Following local anesthesia (Septanest special^®^, noradrenaline 1/100.000, Septodont, Saint Maur des Fossés, France), a crestal incision at the single tooth gap and sulcular incisions at both adjacent teeth were made. Then a full-thickness mucoperiosteal flap was raised. A NobelActive^®^ Wide Platform implant (Nobel Biocare AB, Göteborg, Sweden) was placed in an ideal three-dimensional position [35].

**Figure 1:**
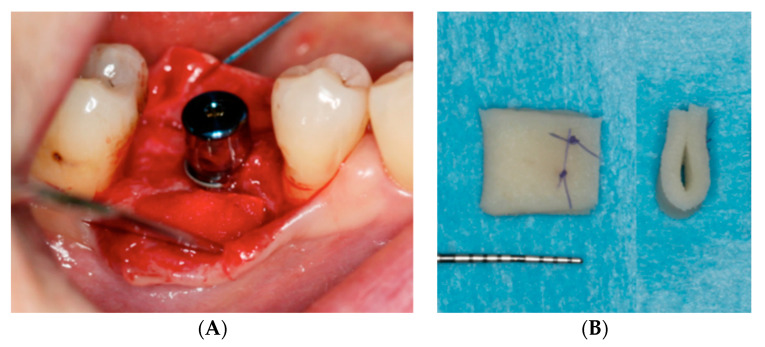
(**A**) Intra-operative view: the porcine-derived collagen matrix (PDCM) (Mucoderm^®^, Botiss gmbh, Berlin, Germany) was trimmed to the desired shape and size and then positioned under the elevated buccal flap. (**B**) In case of advanced horizontal defects, the PDCM was folded in half and secured with resorbable sutures.

Just prior to surgery, a PDCM (Mucoderm^®^, Botiss gmbh, Berlin, Germany) was soaked in sterile saline. After implant installation, it was adapted to the mesiodistal dimension of the defect by trimming with a scalpel. In apico-coronal dimensions, the PDCM was 7 mm. In case of advanced horizontal defects, the 1.5 mm thick matrix was folded in half and secured with resorbable sutures (Serafit^®^ 6/0, Serag-Wiessner, Naila, Germany). As such, a thickness of 3 mm could be reached. Following superficial incision to release muscle tension, the PDCM was positioned under the elevated buccal flap and fixed with one or two sutures (Seralon^®^ 5/0, Serag-Wiessner, Naila, Germany) at the occlusal aspect of the buccal flap (Figure 1).

A healing abutment was connected onto the implant prior to tension-free primary wound closure. Patients were instructed to continue systemic antibiotics (Amoxicillin 1 g, twice/day for 4 days) and to take anti-inflammatory medication when deemed necessary by the patient. Patients rinsed with a 0.12% chlorhexidine solution twice daily for 2 weeks. Then, sutures were removed.

Three months following surgery, permanent crowns were installed. All restorations were screw-retained monolithic Zirconia crowns.

### 2.3. Volumetric Analysis

Alginate impressions (Cavex ColorChange, Haarlem, The Netherlands) were taken at the following time points in each patient: T0 (pre-op), T1 (immediately post-op), T2 (3 months), T3 (1 year) and T4 (3 years). Cast models (Sheraplaster Hartgips White, SHERA Werkstoff-Technologie, Lemförde, Germany) were fabricated within 2 h and optically scanned with a scanner (LS3 scanner, KaVo, Biberach, Germany) creating digital surface models in STL (Surface Tessellation Language) format. The obtained STL files were imported into a digital imaging software program developed to compare surfaces and analyze volumetric changes (SMOP, Swissmeda AG, Zurich, Switzerland).

**Figure 2:**
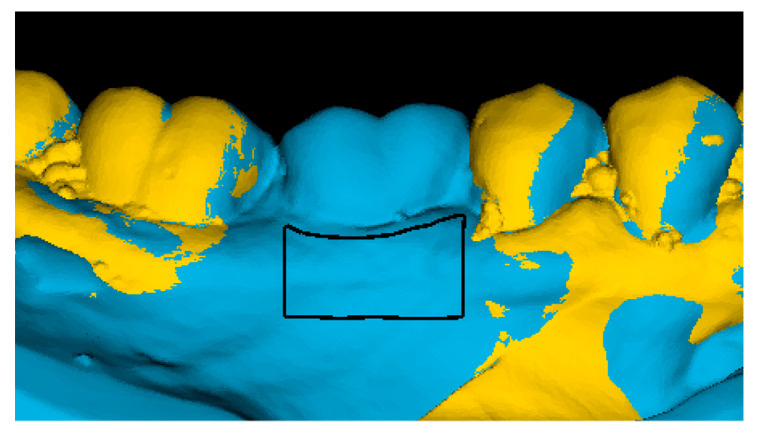
The digital surface model at T0 (yellow) and the digital surface model at T4 (blue) were superimposed. The area of interest (black box), located at the buccal aspect of the grafted area, was determined on the T4 model with the definitive crown in situ.

A study-relevant area of interest (AOI) at the buccal aspect was selected for each augmented site at T4 with the permanent crown in situ. The AOI reached from 0.5 mm below the mucosal margin to 4 mm more apical, determined by a ruler in the software. In the mesiodistal dimension, the AOI reached from the mesial to the distal line angle of the implant crown (Figure 2). The set AOIs varied between patients due to individual anatomic differences but remained fixed in each patient and site over time.

**Figure 3:**
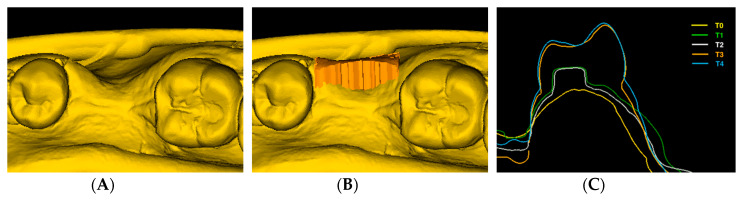
(**A**) Occlusal view of the digital surface model at T0 showing a Seibert Class I defect; (**B**) T0 digital surface model with volumetric changes in the area of interest between T0 and T4; (**C**) Cross- section through the superimposed digital surface models in the center of the grafted area at the different time points.

Each time point was compared to the baseline model (T0–T1, T0–T2, T0–T3 and T0–T4) by superimposing the two models using the best-fit algorithm at the unchanged adjacent tooth surfaces (Figure 3). A mean dimensional change (mm^3^) within the AOI for each patient at T1, T2, T3 and T4 was calculated by the software. As the size of the AOI (mm^2^) differed among patients, the mean volume change per area was converted to a mean linear change in buccal soft tissue profile (BSP) in mm to enable a direct comparison between patients:

(1) $$\Delta d\;\left[mm\right]=\frac{\Delta vol\;\left[mm^{3}\right]}{\Delta area\;\left[mm^{2}\right]}$$

### 2.4. Alveolar Process Deficiency

The alveolar process was evaluated at different time points (T0, T1, T2, T3 and T4) on the basis of an occlusal view of the cast models as described by Fürhauser and co-workers [36]. A score of 0 was given when “obvious alveolar process deficiency” was observed; 1 was given in case of “slight alveolar process deficiency” and 2 was attributed when the alveolar process was not deficient.

### 2.5. Clinical Parameters

#### 2.5.1. Implant Survival

Implant survival was defined as the mere presence of the implant.

#### 2.5.2. Complications

Any biological or technical complication that occurred within the 3-year period was recorded.

#### 2.5.3. Marginal Bone Loss

Marginal bone loss was evaluated on the basis of periapical radiographs taken with the long-cone paralleling technique at implant placement, 1 year and 3 years. The distance from the implant-abutment interface to the first bone-to-implant contact (so-called bone level) was measured at the mesial and distal side of each implant. The implant length was used to calibrate the measurements. Marginal bone loss was calculated at 1 year and 3 years by subtracting bone levels at 3 years and 1 year from bone levels at implant placement. Mesial and distal values were averaged to receive one value per implant.

#### 2.5.4. Probing Depth

Probing depth was recorded at 1 year and 3 years of function. Measurements were performed at four sites (mesio-buccal, buccal, disto-buccal and oral) around the implant and were rounded up to the nearest 0.5 mm. A mean value was calculated per implant.

#### 2.5.5. Plaque and Bleeding on Probing

Plaque and bleeding on probing were recorded at 1 year and 3 years of function. Measurements were performed at four sites (mesio-buccal, buccal, disto-buccal and oral) around the implant. Each site was given a score of 0 or 1 according to the absence or presence of plaque or bleeding on probing, respectively. Both parameters were expressed as a percentage.

### 2.6. Statistical Analysis

All the surgical procedures as well as the clinical measurements and follow-up (implant survival, periapical radiographs, probing depth, plaque and bleeding on probing) were performed by two experienced surgeons (TDB and JC). The measurements for the volumetric analysis, alveolar process deficiency and marginal bone loss were done by an independent examiner (CE). Duplicate measurements were performed for alveolar process deficiency and marginal bone loss by a second independent examiner (EB) on the basis of 20 randomly selected cases (File S1).

Statistical analysis was accomplished in SPSS Statistics 26 (SPSS inc., Chicago, IL, USA) with the patient as the unit of analysis. Descriptive statistics comprised mean values and standard deviations for continuous variables (age, linear change in BSP, marginal bone loss, probing depth, plaque and bleeding on probing) and frequency distributions for categorical variables (gender, jaw, implant length, alveolar process deficiency).

Given a normal distribution of linear changes in BSP, a possible time effect was evaluated using repeated measures ANOVA. In case a significant time effect was found, the paired samples *t*-test was used to compare time points pairwise with a Bonferroni adjustment for multiple comparisons.

Changes in alveolar process deficiency between T0 and T4 were evaluated by means of the Wilcoxon signed-rank test. Kappa statistics were calculated to assess inter-examiner reliability on alveolar process deficiency.

Since violation against a normal distribution was found for marginal bone loss, probing depth, plaque and bleeding on probing, the Wilcoxon signed-rank test was used to compare the 3-year and 1-year results. Inter-examiner reliability on marginal bone loss was assessed using the intraclass correlation coefficient (ICC).

The level of significance was set at 0.05.

## 3. Results

### 3.1. Patients

The study population consisted of 15 patients (10 males, 5 females) with a mean age of 51.4 years (range: 31–79 years). Four wide diameter (5.5 mm) implants were placed in the maxilla, eleven in the mandible. Implant length was 7 mm in 2 patients, 8.5 mm in 6 patients and 10 mm in 7 patients. Of the 15 patients, one dropped out at one-year follow-up due to unwillingness to return despite several contact attempts.

### 3.2. Volumetric Analysis

**Figure 4:**
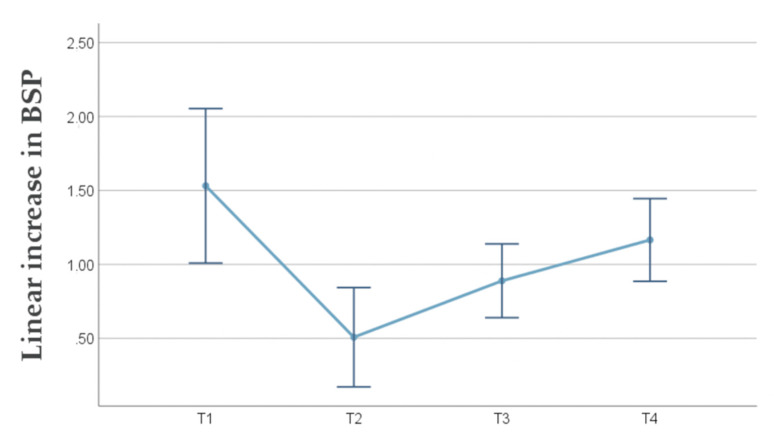
Line chart with the mean linear increase in buccal soft tissue profile (BSP) and 95% confidence interval (CI) at the different time points.

Linear changes in BSP are summarized in Table 1 and Figure 4. A significant time effect was observed (*p* < 0.001). The greatest linear increase in BSP amounted to 1.53 mm and was observed immediately post-surgery (T1) (*p* = 0.001). An obvious shrinkage of 1.02 mm was found at T2 (*p* = 0.001). BSP remained stable between T2 and T3 (*p* = 0.820) and between T3 and T4 (*p* = 0.163). However, between T2 and T4 a significant linear increase in BSP of 0.66 mm was observed (*p* = 0.030). The final linear increase in BSP amounted to 1.17 mm. The latter was significantly different from 0 (*p* < 0.001), indicating that the PDCM was effective in increasing BSP. At three years, a relative horizontal stability of 76.5% was observed since 1.17 mm of the immediate gain of 1.53 mm could be maintained.

### 3.3. Alveolar Process Deficiency

Kappa for inter-examiner reliability on alveolar process deficiency was 0.729 (*p* < 0.001) corresponding to a substantial agreement between duplicate measurements.

**Figure 5:**
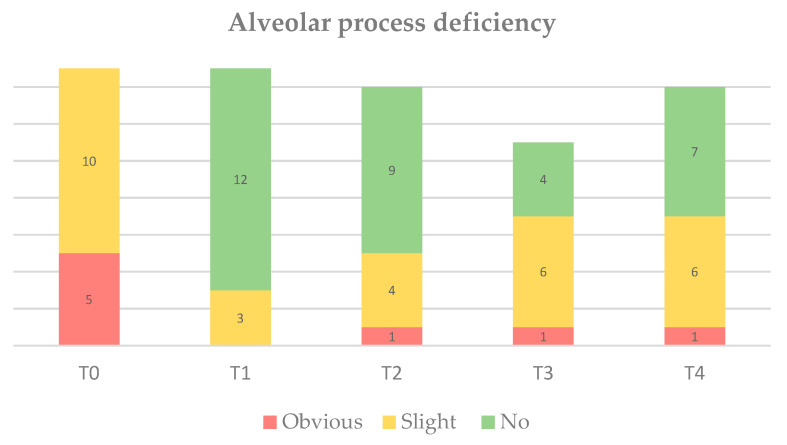
Bar chart on alveolar process deficiency at T0 (pre-op, *n* = 15), T1 (immediately post-op, *n* = 15), T2 (3 months, *n* = 14), T3 (1 year, *n* = 11) and T4 (3 years, *n* = 14).

**Figure 6:**
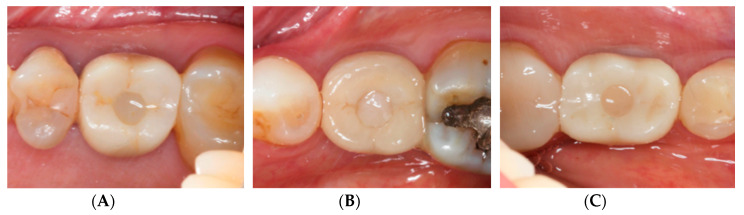
Clinical cases at T4 demonstrating (**A**) no alveolar process deficiency; (**B**) slight alveolar process deficiency; (**C**) obvious alveolar process deficiency.

The results are illustrated in Figure 5. Given the inclusion of Seibert Class I defects at baseline, all patients demonstrated slight-to-obvious alveolar process deficiency prior to surgery. As a result of soft tissue augmentation by means of the PDCM, alveolar process deficiency significantly reduced over time (*p* = 0.004). Interestingly, 50% of patients still demonstrated slight (6/14) or obvious (1/14) alveolar process deficiency at study termination. Figure 6 demonstrates three different clinical cases at T4.

### 3.4. Clinical Parameters

Over the three-year period, all implants survived. With regard to complications, an abscess occurred in two patients at the augmented site during the early stages of healing. These patients took systemic antibiotics for another four days and the symptoms disappeared. There were no other complications over the three-year period.

**Figure 7:**
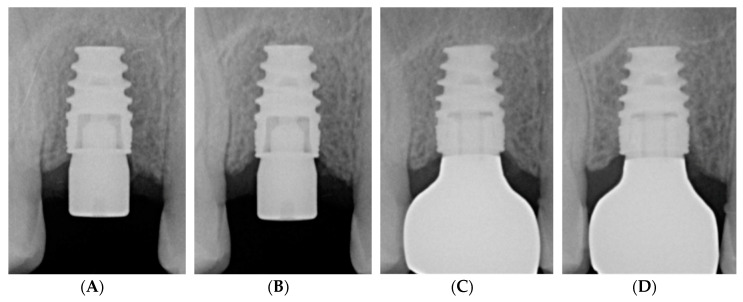
Radiographic follow-up of a NobelActive^®^ Wide Platform implant: (**A**) following implant placement; (**B**) after 3 months; (**C**) after 1 year; (**D**) after 3 years.

Clinical parameters are shown in Table 2. Implants showed low plaque levels, probing depth and bleeding on probing with no significant difference between the one- and three-year data, suggesting healthy clinical conditions until study termination. The ICC on duplicate marginal bone loss registrations amounted to 0.98 (*p* < 0.001), suggesting excellent inter-examiner reliability. Marginal bone loss was not observed. In fact, slight bone gain with borderline significance was found between T3 and T4 (*p* = 0.086). Figure 7 shows periapical radiographs at different time points yielding stable peri-implant conditions.

## 4. Discussion

The primary objective of the present study was to volumetrically evaluate the effectiveness and stability of a newly developed PDCM (Mucoderm^®^, Botiss gmbh, Berlin, Germany) in increasing soft tissue volume at the buccal aspect of molar implant sites. Xenogeneic soft tissue substitutes are currently investigated as alternatives to CTG in order to avoid patient morbidity and complications related to autologous grafting. The results of the present study may add relevant information to the existing knowledge as with three-year data, this study provides the longest follow-up on PDCM for augmenting soft tissue volume.

Soft tissue augmentation is indicated to increase soft tissue volume around dental implants mainly from an aesthetic point of view [1]. As it concerned a newly developed collagen matrix without any evidence in effectiveness and integration at the time this study was initiated, the molar area was chosen. The matrix was not standardized in mesiodistal dimension and thickness. Its dimensions were adapted to the individual site, aiming for a complete fill of the buccal defect. Looking at the results of alveolar process deficiency at T1, this could be achieved in 12 out of 15 patients.

The measurement of soft tissue changes over time is difficult when small alterations occur. In the past, different methods have been used including predominantly transmucosal probing, as well as ultrasonic devices [12,37,38,39] and 2D measurements on CBCT slices [40,41]. The main disadvantage of these methods is that the data only pertain to one specific point of the implant site. More recently, volumetric assessment based on superimposed digital surface models has been developed where the volumetric data pertain to a well-defined area of interest [42]. The volumetric analysis has been shown to be accurate and reproducible [43]. Moreover, the technique is non-invasive and there is no radiation exposure.

The results of the present study demonstrate that the PDCM is effective in increasing buccal soft tissue volume, pointing to a final linear increase in BSP of 1.17 mm. This corresponds to a relative horizontal stability of 76.5% over the three-year period. These findings are in line with two recent clinical studies evaluating the effectiveness of soft tissue volume augmentation by the same PDCM [23,30]. In the study of Zafiropolous et al., 2016 a mean increase in buccal soft tissue thickness of 1.06 mm was measured after six months. The study of Stefanini et al., 2020 reported a mean increase of 1.20 mm after one year. In contrast to the present study, however, both studies used less accurate registration methods based on transmucosal probing with an endodontic instrument or anesthesia needle. In addition, proper baseline registrations are lacking as no measurements were performed immediately post-operative to register the immediate gain.

Apart from the final outcome, the soft tissue changes within the three-year period are important to evaluate. Interestingly, 1.02 mm of the initial volume gain of 1.53 mm got lost after three months (T2), indicating substantial resorption of the matrix. Between T2 and T4, 0.66 mm of that loss was regained. This could imply that the PDCM further remodeled after three months, hereby facilitating connective tissue ingrowth and volume gain. On the other hand, Zafiropoulos et al., 2016 used the same non-cross-linked PDCM and described no remnants after six months which is indicative of fast biodegradation. A more plausible explanation for the tissue gain between T2 and T4 is the installation of the permanent crown, displacing the soft tissues to the buccal aspect. In this regard, it would be meaningful to conduct an RCT comparing the PDCM with no soft tissue augmentation. Such a study would enable an assessment of the true contribution of the matrix to the increase in BSP.

Several clinical studies have been published on alternative collagen matrices for peri-implant soft tissue augmentation. Four studies were found on Mucograft^®^ (Geistlich Pharma AG, Wolhusen, Switzerland) assessing soft tissue thickness changes by transmucosal probing [44,45,46] or ultrasound [39]. The mean increase in buccal soft tissue thickness in these studies ranged from 0.70 mm to 1.16 mm after 3 to 12 months of follow-up. One prospective study was found on OsteoBiol Derma Standard^®^ (Tecnoss, Giaveno, Italy) using the same volumetric measurement method as described in the present study [47]. An initial tissue gain of 1.57 mm was seen two weeks post-augmentation. Of this initial gain, 0.77 mm remained after 24 months pointing to a relative horizontal stability of 49%. Four RCTs from the same research group investigated soft tissue augmentation using Fibro-Gide^®^ (Geistlich Pharma AG, Wolhusen, Switzerland) [48,49,50,51]. Transmucosal probing revealed an initial gain of 1.7 mm with 35% shrinkage after three months and a horizontal stability of 41% three years after crown insertion. Volumetric analysis revealed an initial linear increase in soft tissue thickness of 1.16 mm. After three months, a significant shrinkage of 34% was found. After insertion of the final reconstruction, stable mucosal thickness, or even a slight gain, was observed up to three years. These findings suggest that most resorption takes place in the first three months after augmentation as a result of integration and maturation of the grafting material. Furthermore, the presence of a reconstruction appears to positively affect soft tissue stability. Interestingly, this was also observed in the present study using another PDCM. Altogether, it seems that substantial resorption may be expected during the early stages of healing for all collagen matrices that have been clinically evaluated so far.

Linear increase in BSP may demonstrate clinical effectiveness of a PDCM in thickening the peri-implant mucosa, but this may not necessarily imply that the treatment objective has been reached. The ultimate endpoint in this context is perfect soft tissue convexity at the buccal aspect. In this regard, alveolar process deficiency may be considered an important outcome parameter. A significant reduction in alveolar process deficiency between baseline and three-year follow-up was observed. However, half of the patients still demonstrated slight (6/14) or obvious (1/14) alveolar process deficiency after three years, hereby not reaching the treatment objective. Given this and the high initial resorption rate of collagen matrices, clinicians may want to overcompensate by applying a thicker matrix.

It should be mentioned that mainly short implants were placed in this study due to anatomical restrictions in the posterior segment of the maxilla (sinus floor) and mandible (alveolar nerve). A wide bone condensing implant with variable-thread design (NobelActive^®^ Wide Platform) was used for this purpose. It can be concluded that this implant type rendered healthy peri-implant conditions in the mid-long-term with even a slight bone gain over time. However, these results should be interpreted with some caution since no standardized intra-oral radiographs were taken. These show an overall error of about 0.5 mm when assessing marginal bone level [52], which may explain our results of bone gain.

When interpreting the results of the present study, several limitations should be taken into consideration. First, this is not a randomized controlled study and therefore caution should be taken when comparing the outcome to alternative techniques. In future studies, a comparison with CTG and even with no soft tissue augmentation would be meaningful. Second, a limited number of patients was treated in this study. A limited sample size makes it impossible to detect small changes. Third, conventional impressions are prone to some degree of distortion due to possible volumetric changes of the impression material over time and expansion of the dental stone. In addition, plaster is susceptible to chipping or breakage [53,54,55]. Intra-oral scans are preferred to produce digital surface models. However, at the time this study was initiated, an intra-oral scanner was not available in our center. Alginate impressions were poured in dental stone within 2 h to counteract these possible dimensional alterations. Finally, patient-reported outcome measures are lacking. Sufficiently powered long-term RCTs need to be conducted in the future to evaluate the effectiveness, volume stability and patient-reported outcomes of this collagen matrix as an alternative to CTG in the aesthetic zone.

## 5. Conclusions

The PDCM demonstrated marked resorption during the early stages of healing. Due to the matrix thickening the tissues, and the permanent crown displacing the tissues, 76.5% of the initial increase in BSP could be maintained over a three-year period. Although alveolar process deficiency significantly reduced over time, half of the patients failed to show perfect soft tissue convexity at the buccal aspect after three years.

## Figures and Tables

**Table 1:** Linear increase in buccal soft tissue profile (BSP) at the different time points.

	Mean (SD)	95% CI	*p*-Value
T1	1.53 (0.66)	(1.01; 2.05)	0.001 *
T2	0.51 (0.43)	(0.17; 0.84)	0.001 *
T3	0.89 (0.45)	(0.64; 1.14)	0.820
T4	1.17 (0.52)	(0.89; 1.45)	0.163

* Significantly different to preceding time point.

**Table 2:** Clinical parameters.

Parameter	T3	T4	*p*-Value *
Marginal bone loss (mm) ^§^	**−0.06 (0.85)** *0.05 (−0.10; 0.25)*	**−0.13 (0.70)** *−0.30 (−0.40; 0.05)*	0.086
Probing depth (mm)	**2.5 (0.5)** *2.3 (2.3; 3.3)*	**2.8 (0.9)** *3.0 (2.3; 3.3)*	0.195
Plaque score (%)	**11 (17)** 0 (0; 25)	**20 (21)** *25 (0; 50)*	0.480
Bleeding on probing (%)	**18 (20)** *25 (0; 25)*	**39 (23)** *25 (25; 50)*	0.057

**Bold: Mean (SD);***Italic: Median (IQ range);*^§^ Baseline: implant installation. Negative value indicates bone gain. * Comparison between 1- and 3-year data using the Wilcoxon signed-rank test.

## Supplementary Materials

The following are available online at https://www.mdpi.com/2077-0383/9/5/1568/s1, File S1: Research data.

## References

[1] Wiesner G., Esposito M., Worthington H., Schlee M. (2010). Connective tissue grafts for thickening peri-implant tissues at implant placement. One-year results from an explanatory split-mouth randomised controlled clinical trial. Eur. J. Oral Implantol..

[2] Tan W.L., Wong T.L.T., Wong M.C.M., Lang N.P. (2012). A systematic review of post-extractional alveolar hard and soft tissue dimensional changes in humans. Clin. Oral Implant. Res..

[3] Schneider D., Grunder U., Ender A., Hammerle C.H., Jung R.E. (2011). Volume gain and stability of peri-implant tissue following bone and soft tissue augmentation: 1-year results from a prospective cohort study. Clin. Oral Implant. Res..

[4] Jung R.E., Holderegger C., Sailer I., Khraisat A., Suter A., Hämmerle C.H. (2008). The effect of all-ceramic and porcelain-fused-to-metal restorations on marginal peri-implant soft tissue color: A randomized controlled clinical trial. Int. J. Periodontics Restor. Dent..

[5] Benic G.I., Mokti M., Chen C.J., Weber H.P., Hämmerle C.H., Gallucci G.O. (2012). Dimensions of buccal bone and mucosa at immediately placed implants after 7 years: A clinical and cone beam computed tomography study. Clin. Oral Implant. Res..

[6] Thoma D.S., Buranawat B., Hämmerle C.H., Held U., Jung R.E. (2014). Efficacy of soft tissue augmentation around dental implants and in partially edentulous areas: A systematic review. J. Clin. Periodontol..

[7] Thoma D.S., Mühlemann S., Jung R.E. (2014). Critical soft-tissue dimensions with dental implants and treatment concepts. Periodontology.

[8] Jung R.E., Sailer I., Hammerle C.H., Attin T., Schmidlin P. (2007). In vitro color changes of soft tissues caused by restorative materials. Int. J. Periodontics Restor. Dent..

[9] van Brakel R., Noordmans H.J., Frenken J., de Roode R., de Wit G.C., Cune M.S. (2011). The effect of zirconia and titanium implant abutments on light reflection of the supporting soft tissues. Clin. Oral Implant. Res..

[10] Akcali A., Trullenque-Eriksson A., Sun C., Petrie A., Nibali L., Donos N. (2017). What is the effect of soft tissue thickness on crestal bone loss around dental implants? A systematic review. Clin. Oral Implant. Res..

[11] Thoma D.S., Naenni N., Figuero E., Hämmerle C.H.F., Schwarz F., Jung R.E., Sanz-Sánchez I. (2018). Effects of soft tissue augmentation procedures on peri-implant health or disease: A systematic review and meta-analysis. Clin. Oral Implant. Res..

[12] Eghbali A., Seyssens L., De Bruyckere T., Younes F., Cleymaet R., Cosyn J. (2018). A 5-year prospective study on the clinical and aesthetic outcomes of alveolar ridge preservation and connective tissue graft at the buccal aspect of single implants. J. Clin. Periodontol..

[13] De Bruyckere T., Cosyn J., Younes F., Hellyn J., Bekx J., Cleymaet R., Eghbali A. (2020). A randomized controlled study comparing guided bone regeneration with connective tissue graft to re-establish buccal convexity: One-year aesthetic and patient-reported outcomes. Clin. Oral Implant. Res..

[14] Griffin T.J., Cheung W.S., Zavras A.I., Damoulis P.D. (2006). Postoperative complications following gingival augmentation procedures. J. Periodontol..

[15] Zuhr O., Bäumer D., Hürzeler M. (2014). The addition of soft tissue replacement grafts in plastic periodontal and implant surgery: Critical elements in design and execution. J. Clin. Periodontol..

[16] Cieslik-Wegemund M., Wierucka-Mlynarczyk B., Tanasiewicz M., Gilowski L. (2016). Tunnel Technique with Collagen Matrix Compared with Connective Tissue Graft for Treatment of Periodontal Recession: A Randomized Clinical Trial. J. Periodontol..

[17] Cosgarea R., Juncar R., Arweiler N., Lascu L., Sculean A. (2016). Clinical evaluation of a porcine acellular dermal matrix for the treatment of multiple adjacent class I, II, and III gingival recessions using the modified coronally advanced tunnel technique. Quintessence Int..

[18] Papi P., Pompa G. (2018). The Use of a Novel Porcine Derived Acellular Dermal Matrix (Mucoderm) in Peri-Implant Soft Tissue Augmentation: Preliminary Results of a Prospective Pilot Cohort Study. Biomed Res. Int..

[19] Vincent-Bugnas S., Borie G., Charbit Y. (2018). Treatment of multiple maxillary adjacent class I and II gingival recessions with modified coronally advanced tunnel and a new xenogeneic acellular dermal matrix. J. Esthet. Restor. Dent..

[20] Taba M., Suzuki K., Irie M., Faria P., Messora M., Palioto D., Souza S., Novaes A. (2018). Collagen matrix (Mucoderm) as an alternative for the treatment of gingival recessions. Clin. Oral Implant. Res..

[21] Pietruska M., Skurska A., Podlewski Ł., Milewski R., Pietruski J. (2019). Clinical evaluation of Miller class I and II recessions treatment with the use of modified coronally advanced tunnel technique with either collagen matrix or subepithelial connective tissue graft: A randomized clinical study. J. Clin. Periodontol..

[22] Vellis J., Kutkut A., Al-Sabbagh M. (2019). Comparison of Xenogeneic Collagen Matrix vs. Free Gingival Grafts to Increase the Zone of Keratinized Mucosa Around Functioning Implants. Implant. Dent..

[23] Stefanini M., Rendon A., Zucchelli G. (2020). Porcine-Derived Acellular Dermal Matrix for Buccal Soft Tissue Augmentation at Single Implant Sites: A 1-Year Follow-up Case Series. Int. J. Periodontics Restor. Dent..

[24] Rocchietta I., Schupbach P., Ghezzi C., Maschera E., Simion M. (2012). Soft tissue integration of a porcine collagen membrane: An experimental study in pigs. Int. Periodontics Restor. Dent..

[25] Pabst A.M., Happe A., Callaway A., Ziebart T., Stratul S.I., Ackermann M., Konerding M.A., Willershausen B., Kasaj A. (2014). In vitro and in vivo characterization of porcine acellular dermal matrix for gingival augmentation procedures. J. Periodontal Res..

[26] Barbeck M., Lorenz J., Kubesch A., Bohm N., Booms P., Choukroun J., Sader R., Kirkpatrick C.J., Ghanaati S. (2015). Porcine Dermis-Derived Collagen Membranes Induce Implantation Bed Vascularization Via Multinucleated Giant Cells: A Physiological Reaction?. J. Oral Implantol..

[27] Schmitt C.M., Matta R.E., Moest T., Humann J., Gammel L., Neukam F.W., Schlegel K.A. (2016). Soft tissue volume alterations after connective tissue grafting at teeth: The subepithelial autologous connective tissue graft versus a porcine collagen matrix - a pre-clinical volumetric analysis. J. Clin. Periodontol..

[28] Pabst A.M., Lehmann K.M., Walter C., Kruger M., Stratul S.I., Kasaj A. (2016). Influence of porcine-derived collagen matrix on endothelial progenitor cells: An in vitro study. Odontology.

[29] Puisys A., Zukauskas S., Kubilius R., Barbeck M., Razukevicius D., Linkeviciene L., Linkevicius T. (2019). Clinical and Histologic Evaluations of Porcine-Derived Collagen Matrix Membrane Used for Vertical Soft Tissue Augmentation: A Case Series. Int. J. Periodontics Restor. Dent..

[30] Zafiropoulos G.G., Deli G., Hoffmann O., John G. (2016). Changes of the peri-implant soft tissue thickness after grafting with a collagen matrix. J. Indian Soc. Periodontol..

[31] O’Leary T.J., Drake R.B., Naylor J.E. (1972). The plaque control record. J. Periodontol..

[32] Seibert J.S. (1983). Reconstruction of deformed, partially edentulous ridges, using full thickness onlay grafts. Part I. Technique and wound healing. Compend. Contin. Educ. Dent..

[33] Agha R.A., Fowler A.J., Rajmohan S., Barai I., Orgill D.P. (2016). Preferred reporting of case series in surgery; the PROCESS guidelines. Int. J. Surg..

[34] Agha R.A., Borrelli M.R., Farwana R., Koshy K., Fowler A.J., Orgill D.P. (2018). The PROCESS 2018 statement: Updating Consensus Preferred Reporting of CasE Series in Surgery (PROCESS) guidelines. Int. J. Surg..

[35] Buser D., Martin W., Belser U.C. (2004). Optimizing esthetics for implant restorations in the anterior maxilla: Anatomic and surgical considerations. Int. J. Oral Maxillofac. Implant..

[36] Furhauser R., Florescu D., Benesch T., Haas R., Mailath G., Watzek G. (2005). Evaluation of soft tissue around single-tooth implant crowns: The pink esthetic score. Clin. Oral Implant. Res..

[37] De Bruyckere T., Eghbali A., Younes F., De Bruyn H., Cosyn J. (2015). Horizontal stability of connective tissue grafts at the buccal aspect of single implants: A1-year prospective case series. J. Clin. Periodontol..

[38] Eghbali A., De Bruyn H., Cosyn J., Kerckaert I., Van Hoof T. (2016). Ultrasonic Assessment of Mucosal Thickness around Implants: Validity, Reproducibility, and Stability of Connective Tissue Grafts at the Buccal Aspect. Clin. Implant. Dent. Relat. Res..

[39] Puzio M., Blaszczyszyn A., Hadzik J., Dominiak M. (2018). Ultrasound assessment of soft tissue augmentation around implants in the aesthetic zone using a connective tissue graft and xenogeneic collagen matrix – 1-Year randomised follow-up. Ann. Anat..

[40] De Bruyckere T., Eeckhout C., Eghbali A., Younes F., Vandekerckhove P., Cleymaet R., Cosyn J. (2018). A randomized controlled study comparing guided bone regeneration with connective tissue graft to re-establish convexity at the buccal aspect of single implants: A one-year CBCT analysis. J. Clin. Periodontol..

[41] Seyssens L., Eghbali A., Christiaens V., De Bruyckere T., Doornewaard R., Cosyn J. (2019). A one-year prospective study on alveolar ridge preservation using collagen-enriched deproteinized bovine bone mineral and saddle connective tissue graft: A cone beam computed tomography analysis. Clin. Implant. Dent. Relat. Res..

[42] Giannobile W., Lang N., Tonetti M. (2014). Chapter Analytical methods. Osteology Guidelines for Oral and Maxillofacial Regeneration. Clinical Research.

[43] Windisch S.I., Jung R.E., Sailer I., Studer S.P., Ender A., Hammerle C.H. (2007). A new optical method to evaluate three-dimensional volume changes of alveolar contours: A methodological in vitro study. Clin. Oral Implant. Res..

[44] Froum S.J., Khouly I., Tarnow D.P., Froum S., Rosenberg E., Corby P., Kye W., Elian N., Schoor R., Cho S.C. (2015). The use of a xenogeneic collagen matrix at the time of implant placement to increase the volume of buccal soft tissue. Int. J. Periodontics Restor. Dent..

[45] Schallhorn R.A., McClain P.K., Charles A., Clem D., Newman M.G. (2015). Evaluation of a porcine collagen matrix used to augment keratinized tissue and increase soft tissue thickness around existing dental implants. Int. J Periodontics Restor. Dent..

[46] Cairo F., Barbato L., Tonelli P., Batalocco G., Pagavino G., Nieri M. (2017). Xenogeneic collagen matrix versus connective tissue graft for buccal soft tissue augmentation at implant site. A randomized, controlled clinical trial. J. Clin. Periodontol..

[47] Fischer K.R., Testori T., Wachtel H., Muhlemann S., Happe A., Del Fabbro M. (2019). Soft tissue augmentation applying a collagenated porcine dermal matrix during second stage surgery: A prospective multicenter case series. Clin. Implant. Dent. Relat. Res..

[48] Thoma D.S., Zeltner M., Hilbe M., Hammerle C.H., Husler J., Jung R.E. (2016). Randomized controlled clinical study evaluating effectiveness and safety of a volume-stable collagen matrix compared to autogenous connective tissue grafts for soft tissue augmentation at implant sites. J. Clin. Periodontol..

[49] Zeltner M., Jung R.E., Hammerle C.H., Husler J., Thoma D.S. (2017). Randomized controlled clinical study comparing a volume-stable collagen matrix to autogenous connective tissue grafts for soft tissue augmentation at implant sites: Linear volumetric soft tissue changes up to 3 months. J. Clin. Periodontol..

[50] Huber S., Zeltner M., Hammerle C.H.F., Jung R.E., Thoma D.S. (2018). Non-interventional 1-year follow-up study of peri-implant soft tissues following previous soft tissue augmentation and crown insertion in single-tooth gaps. J. Clin. Periodontol..

[51] Thoma D.S., Gasser T.J.W., Jung R.E., Hammerle C.H.F. (2020). Randomized controlled clinical trial comparing implant sites augmented with a volume-stable collagen matrix or an autogenous connective tissue graft: 3-year data after insertion of reconstructions. J. Clin. Periodontol..

[52] De Smet E., Jacobs R., Gijbels F., Naert I. (2002). The accuracy and reliability of radiographic methods for the assessment of marginal bone level around oral implants. Dentomaxillofac. Radiol..

[53] Abizadeh N., Moles D.R., O’Neill J., Noar J.H. (2012). Digital versus plaster study models: How accurate and reproducible are they?. J. Orthod..

[54] Todd J.A., Oesterle L.J., Newman S.M., Shellhart W.C. (2013). Dimensional changes of extended-pour alginate impression materials. Am. J. Orthod. Dentofac. Orthop..

[55] Tomita Y., Uechi J., Konno M., Sasamoto S., Iijima M., Mizoguchi I. (2018). Accuracy of digital models generated by conventional impression/plaster-model methods and intraoral scanning. Dent. Mater. J..

